# Brain mitochondrial oxidative metabolism during and after cerebral hypoxia–ischemia studied by simultaneous phosphorus magnetic-resonance and broadband near-infrared spectroscopy^☆^

**DOI:** 10.1016/j.neuroimage.2013.08.016

**Published:** 2014-11-15

**Authors:** A. Bainbridge, I. Tachtsidis, S.D. Faulkner, D. Price, T. Zhu, E. Baer, K.D. Broad, D.L. Thomas, E.B. Cady, N.J. Robertson, X. Golay

**Affiliations:** a Medical Physics and Bioengineering, UCLH NHS Foundation Trust, London NW1 2BU, UK; b Medical Physics and Bioengineering, University College London, WC1E 6BT, UK; c Institute for Women's Health, University College London, WC1E 6AU, UK; d Institute of Neurology, University College London, London WC1N 3BG, UK

**Keywords:** NIRS, Cytochrome-*c*-oxidase, MRS, Phosphorus, ^31^P, Hypoxia–ischemia

## Abstract

**Background:**

Multimodal measurements combining broadband near-infrared spectroscopy (NIRS) and phosphorus magnetic resonance spectroscopy (^31^P MRS) assessed associations between changes in the oxidation state of cerebral mitochondrial cytochrome-*c*-oxidase (Δ[_ox_CCO]) and ^31^P metabolite peak-area ratios during and after transient cerebral hypoxia–ischemia (HI) in the newborn piglet.

**Methods:**

Twenty-four piglets (aged < 24 h) underwent transient HI (inspired oxygen fraction 9% and bilateral carotid artery occlusion for ~ 20 min). Whole-brain ^31^P MRS and NIRS data were acquired every minute. Inorganic phosphate (Pi)/epp, phosphocreatine (PCr)/epp, and total nucleotide triphosphate (NTP)/epp were measured by ^31^P MRS and were plotted against Δ[_ox_CCO] during HI and recovery (epp = exchangeable phosphate pool = Pi + PCr + 2γ-NTP + β-NTP).

**Results:**

During HI Δ[_ox_CCO], PCr/epp and NTP/epp declined and Pi/epp increased. Significant correlations were seen between ^31^P ratios and Δ[_ox_CCO]; during HI a threshold point was identified where the relationship between Δ[_ox_CCO] and both NTP/epp and Pi/epp changed significantly. Outcome at 48 h related to recovery of Δ[_ox_CCO] and ^31^P ratios 1 h post-HI (survived: 1-h NTP/epp 0.22 ± 0.02, Δ[_ox_CCO] − 0.29 ± 0.50 μM; died: 1-h NTP/epp 0.10 ± 0.04, Δ[_ox_CCO] − 2.41 ± 1.48 μM).

**Conclusions:**

Both lowered Δ[_ox_CCO] and NTP/epp 1 h post-HI indicated mitochondrial impairment. Animals dying before 48 h had slower recovery of both Δ[_ox_CCO] and ^31^P ratios by 1 h after HI.

## Highlights

• ^31^P MRS correlated with broadband NIRS during and after transient hypoxia–ischemia.
• A double-linear model best describes NTP/epp vs Δ[_ox_CCO] during HI.
• Threshold point in double-linear model interpreted as cessation of ATP manufacture.
• Poor outcome correlates with weaker recovery of both ^31^P MRS ratios and Δ[_ox_CCO].
• Mitochondrial dysfunction may be the cause of reduction of Δ[_ox_CCO].

## Introduction

Neonatal encephalopathy following perinatal hypoxia–ischemia (HI) is associated with high rates of mortality and morbidity worldwide (Kurinczuk et al., 2010). Although successful treatment strategies such as therapeutic hypothermia (Tagin et al., 2012) have been developed and are in use in the developed world, there is still an urgent need for novel therapies to improve clinical outcomes. There is also a need to detect those individuals at most risk of brain injury and who may benefit from adjunct therapies or redirection of clinical care.

In human infants, phosphorus magnetic resonance spectroscopy (^31^P MRS) can be used to study the evolution of cerebral energetic metabolism following intra-partum HI (Hope et al., 1984). In infants with eventual adverse outcome, despite adequate oxygenation and circulation, phosphocreatine (PCr) and nucleotide triphosphate (NTP—mainly adenosine triphosphate, ATP) decline and inorganic phosphate (Pi) increases in the first days of life (Cady et al., 1997). These metabolic changes are termed “secondary energy failure” (SEF) on the basis that they followed primary intra-partum cerebral energy generation impairment (resulting in transiently reduced PCr and NTP and increased Pi), which resolved following resuscitation (Lorek et al., 1994). Since the 1980s there have been few studies using ^31^P MRS as outcome biomarkers in babies following asphyxia, as the ^31^P MRS facility is not routinely available on clinical scanners, but the decline in the brain energy measured in this manner in the days and hours after birth strongly correlates with neurodevelopmental outcome and head growth (Azzopardi et al., 1989). The main MRS biomarker now used in babies to predict outcome and as a surrogate outcome measure in clinical neuroprotection trials (e.g. TOBY Xenon) is the proton magnetic resonance spectroscopy (^1^H MRS) thalamic lactate/N acetyl aspartate (NAA) peak area ratio (Thayyil et al., 2010). Lactate/NAA correlates with the severity of PCr/Pi decline. ^1^H MRS is attractive as it is practical and easy, using the same nucleus as MR imaging, and provides a higher resolution with smaller voxel sizes compared with ^31^P MRS. Lactate/NAA is currently used as a surrogate outcome measure in phase II clinical trials, see e.g.: www.ClinicalTrials.gov identifier: NCT00934700

Broadband near-infrared spectroscopy (NIRS) provides brain energetics information complementary to MRS. In adult humans, NIRS has measured concentration changes in oxygenated haemoglobin (HbO_2_), de-oxygenated haemoglobin (HHb), and the oxidation state of cytochrome-*c*-oxidase (Δ[_ox_CCO]) (Tachtsidis et al., 2009, Tachtsidis et al., 2011, Tisdall et al., 2007, Tisdall et al., 2008a). CCO is the terminal electron acceptor of the mitochondrial electron transfer chain and therefore plays a crucial role in cellular O_2_ utilization and ATP synthesis (Richter and Ludwig, 2003). NIRS can be a cotside tool for human neonates and several NIRS instruments monitoring only tissue oxygen saturation (SO_2_) are approved for clinical use (Wolf et al., 2007). However, these cerebral oximeters have attracted specific criticisms: (i) SO_2_ alone is not a robust indicator of sufficient tissue oxygen delivery (Weiss et al., 2005); (ii) the NIRS measurement has poor reliability compared with SO_2_ in jugular venous blood (Nagdyman et al., 2005); and (iii) the absolute oxygenation level is relatively poorly determined (Greisen et al., 2011). However, broadband NIRS enables Δ[_ox_CCO] monitoring and measuring Δ[_ox_CCO] during recovery from HI may provide additional clinical utility.

Our study uses an established piglet model of transient perinatal HI (Faulkner et al., 2011, O'Brien et al., 2006, Robertson et al., 2012a, Robertson et al., 2012b) utilizing ^31^P MRS to quantify cerebral energy metabolism before, during and after transient HI. Measurement of Δ[_ox_CCO] in parallel with ^31^P MRS has been previously reported in the piglet. Cooper and Springett reported measurement of Δ[_ox_CCO] contemporaneously with ^31^P MRS in a single piglet during HI and up to 40 hof recovery (Cooper and Springett, 1997). However, ^31^P MRS temporal resolution in that work was low, comprising 3 measurements covering baseline and HI and 4 during the first hour of recovery. Springett et al. (2000a) reported measurements made during brief (105 s) anoxias. Temporal resolution was better but anoxia was insufficient to reduce NTP generation.

In this current study, our goal was to ascertain how Δ[_ox_CCO] correlates with changes in energy metabolism during HI and during early recovery after reperfusion and re-oxygenation. We achieved this using broadband NIRS and ^31^P MRS simultaneously to provide continuous measurements every 1 min. Our hypothesis was that the extent of recovery of Δ[_ox_CCO] and ^31^P MRS metabolite ratios would correlate during early recovery post HI.

## Methods

### Subjects and maintenance

All animal experiments were performed under UK Home Office Guidelines (Animals [Scientific procedures] act, 1986). Twenty-four male term-born piglets were anesthetized and surgically prepared (aged < 24 h) as described previously (Lorek et al., 1994). Briefly, piglets were sedated with intramuscular midazolam (0.2 mg/kg) and arterial O_2_ saturation (SpO_2_) was monitored (Nonin Medical, USA). Isoflurane anesthesia (4% (vol/vol)) was initially given through a facemask to facilitate tracheostomy and intubation and was maintained throughout (3% during surgery and 2% otherwise). Piglets had mechanical ventilation adjusted to maintain partial pressures of oxygen (PaO_2_) and carbon dioxide (PaCO_2_) at 8–13 kPa and 4.5–6.5 kPa, respectively allowing for arterial-blood temperature correction.

An umbilical venous catheter was inserted for infusion of maintenance fluids (10% dextrose, 60 ml/kg/day), fentanyl (3–6 μg/kg/h), and antibiotics (benzylpenicillin 50 mg/kg and gentamicin 2.5 mg/kg, every 12 h). An umbilical arterial catheter was inserted for continuous heart rate (HR) and mean arterial blood pressure (MABP) monitoring and intermittent measurement of blood PaO_2_, PaCO_2_, pH, electrolytes, glucose and lactate (Abbot Laboratories, UK). Bolus infusions of colloid (Gelofusin, B Braun Medical Ltd., Emmenbrucke, Switzerland) and inotropes maintained MABP at > 40 mm Hg. All animals received continuous physiological monitoring (SA Instruments, New York, USA) and intensive life support throughout experimentation. Arterial line clotting was prevented by continually infusing 0.9% saline (Baxter, Deerfield, IL;1 ml/h) with heparin sodium added (1 IU/ml).

Both common carotid arteries were surgically isolated at the level of the fourth cervical vertebra and encircled by remotely controlled vascular occluders (OC2A, In Vivo Metric, Healdsburg, California, USA). Then piglets were positioned prone in a plastic pod and the head immobilised in a stereotactic frame which included NIRS optodes placed against the sides of the head. A 7 cm × 5 cm elliptical transmit–receive MRS surface coil tuned to the ^31^P resonant frequency (51.6 MHz) was positioned on top of the head. NIRS and MRS data were acquired before, during and up to 60 min after HI. MRS data were also acquired 24 h and 48 h post HI.

### Broadband NIRS

For NIRS we used an “in house” constructed broadband spectrometer previously used for other studies of piglet brain (Cooper and Springett, 1997, Springett et al., 2000a), and brain injury patients (Tisdall et al., 2008a). Light from a stabilized tungsten halogen source was filtered with 610-nm long-pass and heat-absorbing filters, and transmitted to an optode secured stereo-tactically against the left side of the head via a 3.3-mm diameter glass, 7 m long optical fibre bundle. This setup did not cause any heating of the tissue or any other diverse effects. Light incident on the detector optode (right side of head) was focused via an identical fibre bundle onto the 400-μm entrance slit of a 0.27-m spectrograph (270M, Instruments SA, France) with a 300-g/mm grating blazed at 1000 nm. Optodes were co-linear over the ventromedial/temporal region with a line passing through the brain centre. Spectra between 650 nm and 980 nm were continuously acquired every 1 min on a cooled-charge coupled device detector (Wright Instruments, United Kingdom) with the shutter open, giving a signal of 10,000–30,000 electrons per digital conversion at 800 nm and about 1 nm dispersion per pixel and an approximate spectral resolution of less than 5 nm. A reference spectrum was acquired and changes (Δ) in the brain concentrations of HbO_2_, HHb, and oxidised CCO were determined using the UCLn algorithm (Matcher et al., 1995) after correction for the wavelength dependence of path-length (Essenpreis et al., 1993). The optical set-up, optical algorithm and experimental methodology did not allow us to measure the absolute baseline oxidation state of CCO. The optical path length for the 840-nm water absorption feature was obtained by fitting the second differential of the attenuation spectra to the second differential of the *in vitro* water signal between 800 and 880 nm and assuming 85% cerebral water content (Matcher et al., 1994). Changes in haemoglobin difference (Δ[Hb_diff_] = Δ[HbO_2_] − Δ[HHb]; indexing changes in brain oxygenation) and changes in total haemoglobin (Δ[Hb_tot_] = Δ[HbO_2_] + Δ[HHb]; indexing cerebral blood volume) were also calculated. The measurements of Δ[_ox_CCO], Δ[HbO_2_] and Δ[HHb] are defined to be zero at the point of first measurement. Thus their values over the course of the experiment represent the aggregate change with respect to this first measurement.

### ^31^P MRS

Simultaneously with NIRS, whole-brain ^31^P MRS was acquired with 1 min resolution using a 9.4 T Agilent spectrometer and non-localised single-pulse surface-coil acquisition (repetition time 10 s, 6 summed acquisitions per spectrum). MRS data were analysed using AMARES (Vanhamme et al., 1997) as implemented in the jMRUI software (van den Boogaart, 1997). Prior knowledge of the NTP multiplet structure was used (fitting doublets to α- and γ-NTP and a triplet to β-NTP) but no assumption was made as to multiplet relative sizes. NTP is predominately ATP and the latter contributes approximately 70% of the NTP signal e.g. in the rat pup (Mandel and Edel-Harth, 1966). Thus NTP changes during this experiment predominately reflected ATP changes. Pi was fitted using 4 separate components and PCr with a single component. The following peak-area ratios were calculated: Pi/epp, PCr/epp, and NTP/epp where epp = exchangeable phosphate pool = Pi + PCr + 2γ-NTP + β-NTP.

### Hypoxia–ischemia

Baseline NIRS and MRS data were recorded for 10 min before HI. HI was induced inside the MR scanner by remotely inflating the vascular occluders around both common-carotid arteries, and simultaneously reducing fractional inspired (Fi) O_2_ to 9% (vol/vol). During HI the β-NTP peak height was continuously monitored using in-house Matlab (Mathworks) software. The period from the start of HI to when β-NTP had fallen to 40% of its baseline height is defined as “Initial HI”. At the end of Initial HI FiO_2_ was titrated to maintain β-NTP peak height between 30% and 40% of baseline for 12.5 min (the “Titration” period). At the end of Titration the occluders were deflated and FiO_2_ normalised (the “Recovery” period).

### Statistics

When grouped data are used results are mean (standard deviation). Data were tested for normality prior to parametric statistics using an Anderson–Darling normality test with a null hypothesis that the data were normally distributed. Where data were normally distributed, analysis of variance (ANOVA) compared grouped data with the significance of any difference being determined by a Student t-test. Where data were not normally distributed, a Wilcoxon rank sum test was used instead. Significance was assumed for p < 0.05.

^31^P MRS ratios were plotted against Δ[_ox_CCO]. During Initial HI and Titration plots were fitted with a linear function as a first level of analysis. Additionally, in order to model any threshold change at a particular Δ[_ox_CCO] the same plots were fitted with a double-linear function of the following form:

(1) $$\begin{array}{cc} y=\mathrm{ax}+b & :x<A\\ y=\mathrm{cx}+d & :x>A\\ \mathrm{ax}+b=\mathrm{cx}+d & :x=A \end{array}$$

where y = ^31^P MRS ratio and x = Δ[_ox_CCO]; A is Δ[_ox_CCO] at the point of gradient change. The final condition ensures a continuous function (however, note that it is not continuously derivable). For display plots were transposed so point A was the origin. For recovery comparisons plots were fitted with only a linear function. Δ[_ox_CCO] and ^31^P MRS ratios were expected to show heterogeneous recovery as measurements during this period are an average over diverse populations of cells recovering at different rates. The measured relationships between Δ[_ox_CCO] and ^31^P MRS ratios were therefore not expected to reflect the relationships in a single-cell and thus the double linear model was not used for the recovery data [see the section “Interpretation of Results”].

## Results

### Physiology

Baseline 10-min average physiology measurements were all normal for a newborn piglet. Table 1 shows mean physiology measurements over all subjects at baseline, end of Initial HI and 10 min and 60 min into Recovery. The mean duration of HI was 27.5 (3.8) min. During Initial HI SpO_2_ was significantly reduced due to decreased FiO_2_. MABP decreased significantly during HI and then normalised during Recovery. HR was elevated during HI and early Recovery. Rectal temperature was unchanged.

**Table 1:** Mean physiological data measured at baseline, Initial HI end, 10 min into Recovery and 60 min into Recovery. SpO_2_: arterial oxygen saturation, HR: heart rate, MABP: mean arterial blood pressure.

Time	Rectal temperature (°C)	SpO_2_ (%)	HR (beats/min)	MABP (mm Hg)
Baseline	38.5 (0.8)	96.8 (2.3)	152 (19)	48.1 (5.6)
Initial HI end	38.1 (0.4)	44.1 (10.8)^b^	166 (23)^a^	32.6 (8.1)^b^
10 min recovery	38.1 (0.4)	94.4 (2.9)	166 (25)^a^	47.2 (11.5)
60 min recovery	38.3 (0.6)	95.8 (3.2)	159 (30)	47.4 (8.5)

Paired T-test vs baseline: ^a^p < 0.05

Paired T-test vs baseline: ^b^p < 0.01

### Temporal NIRS and ^31^P MRS measurements

**Fig. 1:**
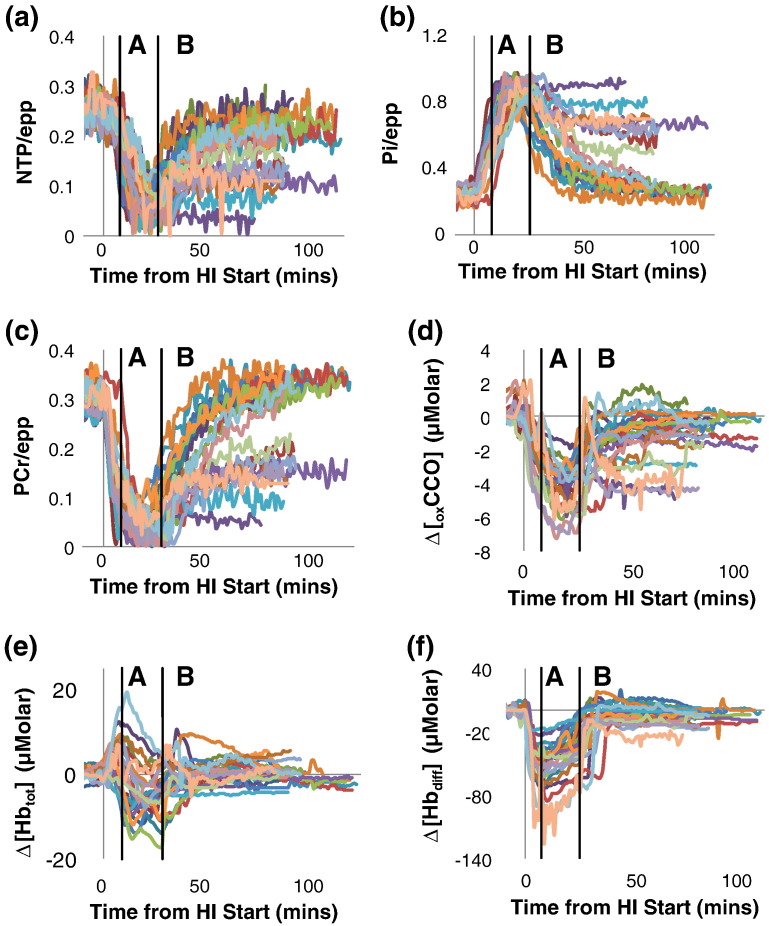
^31^P MRS metabolite ratios and NIRS measurements plotted against time for each piglet: a) NTP/epp, b) Pi/epp, c) PCr/epp, d) Δ[_ox_CCO], e) Δ[Hb_tot_] and f) Δ[Hb_diff_]. Data from all animals are shown. The mean time at which point A_NTP_ is reached is shown in each plot by the line marked A (see the section “Relationships between Δ[oxCCO] and MRS measures during initial HI and titration” and Fig. 2). The mean time at which HI was ended and the recovery period began is shown on each plot by the line marked B.

Fig. 1 shows temporal NIRS and ^31^P MRS measurements for each piglet. During HI Δ[Hb_tot_] generally increased briefly (mean maximum during Initial HI period 5.2 (4.7) (range − 0.1 to 19.3) μM) before decreasing later (mean minimum during Titration period − 6.7 (5.0) (range − 17.4 to 0.3) μM). During Recovery Δ[Hb_tot_] returned to baseline or slightly less (mean maximum during Recovery period 3.1 (3.4), range − 4.2 to 10.5 μM). In all piglets Δ[Hb_diff_] declined at HI initiation (mean minimum during Initial HI period − 59 (24) (range − 127 to − 19) μM) with eventual regain towards baseline during Recovery (mean maximum during Recovery period 2.0 (8.2), range − 10.6 to 19.5, μM). There were no overall relationships between Δ[Hb_tot_] and Δ[Hb_diff_] at 1 h and NTP/epp at 1 h, 24 h or 48 h after HI.

Baseline ^31^P MRS ratios and Δ[_ox_CCO] are in Table 2. Δ[_ox_CCO] fell rapidly at the start of HI minimising during Titration (mean minimum during Titration period − 4.5 (1.4) (range − 2.1 to 7.2) μM) and then increasing during Recovery: however, baseline was not reattained in some piglets. During HI, PCr/epp decreased (mean minimum during Initial HI and Titration periods 0.02 (0.02) (range 0.001 to 0.08)) and Pi/epp increased (mean maximum during Initial HI and Titration periods 0.91 (0.04), (range 0.81 to 0.98)). During HI NTP/epp briefly remained near baseline before decreasing (mean minimum during Initial HI and Titration periods 0.04 (0.02) (range 0.004 to 0.09)) once PCr/epp had declined substantially.

**Table 2:** Mean ^31^P MRS ratios and Δ[_ox_CCO] measured during 10 min of data acquisition at baseline. Δ[_ox_CCO] is defined to be zero at the first measurement. Thus the value for Δ[_ox_CCO] represents the mean change during the baseline measurement period (see the section “Broadband NIRS”). Data are presented stratified according to outcome at 48 h (survived or died) and without stratification (survived plus died). No significant differences were seen between the outcome groups at baseline.

Outcome Group	PCr/epp	Pi/epp	NTP/epp	Δ[_ox_CCO] (μM)
Survived to 48 h	0.31 (0.02)	0.24 (0.03)	0.25 (0.02)	0.09 (0.38)
Died pre 48 h	0.29 (0.02)	0.26 (0.03)	0.25 (0.02)	0.25 (0.60)
Survived plus died	0.30 (0.02)	0.25 (0.03)	0.25 (0.02)	0.15 (0.47)

### Outcomes

^31^P MRS ratios and Δ[_ox_CCO] 1 h into Recovery and NTP/epp 24 h and 48 h after HI are in Table 3. Piglets were grouped according to whether they: (a) died (including termination) before 48 h due to physiological deterioration consequential to HI; or (b) survived to 48 h. Nine subjects died before 48 h: 4 had cardiac arrest between 24 and 48 h and 1 before 24 h; the remaining 4 were terminated within 3 h of HI because NTP/epp did not recover to baseline (experiment continuation would have been unethical). The remaining 15 piglets all survived to 48 h.

**Table 3:** ^31^P MRS ratios and Δ[_ox_CCO] measured at 1 h into Recovery and also NTP/epp measured at 24 h and 48 h. Data are stratified according to outcome at 48 h (survived or died). Data are given for each animal along with outcome-group means. Group means were tested for differences at 1 h, 24 h and 48 h compared to baseline values and for differences at 1 h between the outcome groups. Where animals had died prior to MRS measurement at 24 h or 48 h, the table entry is left blank.

Subject number	1 h after HI				Outcome	
	PCr/epp	Pi/epp	NTP/epp	Δ[_ox_CCO] (μM)	NTP/epp24 h	NTP/epp48 h
Survived to 48 h						
1	0.34	0.27	0.19	0.14	0.26	0.25
2	0.34	0.22	0.23	0.20	0.25	0.24
3	0.33	0.28	0.21	− 0.49	0.23	0.24
4	0.33	0.31	0.19	− 1.07	0.24	0.24
5	0.31	0.28	0.22	− 0.33	0.24	0.24
8	0.34	0.29	0.20	0.04	0.22	0.24
10	0.31	0.28	0.23	− 0.59	0.21	0.22
11	0.30	0.33	0.22	− 0.57	0.24	0.23
12	0.30	0.28	0.25	0.88	0.22	0.23
13	0.32	0.27	0.24	− 0.15	0.24	0.21
14	0.32	0.29	0.22	− 0.54	0.25	0.23
16	0.29	0.35	0.19	− 0.69	0.24	0.24
19	0.32	0.31	0.21	0.08	0.24	0.23
21	0.30	0.31	0.23	− 0.89	0.22	0.21
23	0.32	0.30	0.21	− 0.36	0.22	0.12
Survivor group means	0.32 (0.02)	0.29^b^ (0.03)	0.22^b^ (0.02)	− 0.29^d^ (0.50)	0.23^b^ (0.01)	0.22^b^ (0.03)
Died before 48 h						
6	0.15	0.65	0.11	− 1.48	0.02	−
7	0.09	0.79	0.07	− 2.88	0.04	–
9	0.16	0.63	0.12	− 1.23	–	–
15	0.14	0.71	0.09	− 1.79	0.04	–
17	0.21	0.50	0.16	− 1.71	–	–
18	0.15	0.65	0.12	− 4.34	0.14	–
20	0.19	0.57	0.13	− 0.19	–	–
22	0.05	0.91	0.03	− 4.35	–	–
24	0.13	0.70	0.10	− 3.76	–	–
Died group means	0.14^b,c^ (0.05)	0.68^e,f^ (0.12)	0.10^e,f^ (0.04)	− 2.41^e,f^ (1.48)	–	–

Paired t-test vs baseline: ^a^p < 0.05, ^b^p < 0.01.

T-test vs 1 h recovery in survivors: ^c^p < 0.01

Wilcoxon Rank Sums test vs baseline: ^d^p < 0.05, ^e^p < 0.01

Wilcoxon Rank Sums test vs 1 h recovery in survivors: ^f^p < 0.01

In piglets surviving to 48 h, by 1 h into Recovery PCr/epp had recovered to baseline; Pi/epp remained higher and NTP/epp and Δ[_ox_CCO] lower than at baseline (Table 3). In those dying before 48 h, 1 h into Recovery PCr/epp, Pi/epp, NTP/epp and Δ[_ox_CCO] were all significantly different from their baselines and from the 1-h recovery values for those surviving to 48 h. The was no significant difference in either Δ[Hb_tot_] or Δ[Hb_diff_] between the outcome groups at 1 h recovery (t-test, p > 0.9 and p > 0.1 respectively).

### Relationships between Δ[_ox_CCO] and MRS measures during Initial HI and Titration

**Fig. 2:**
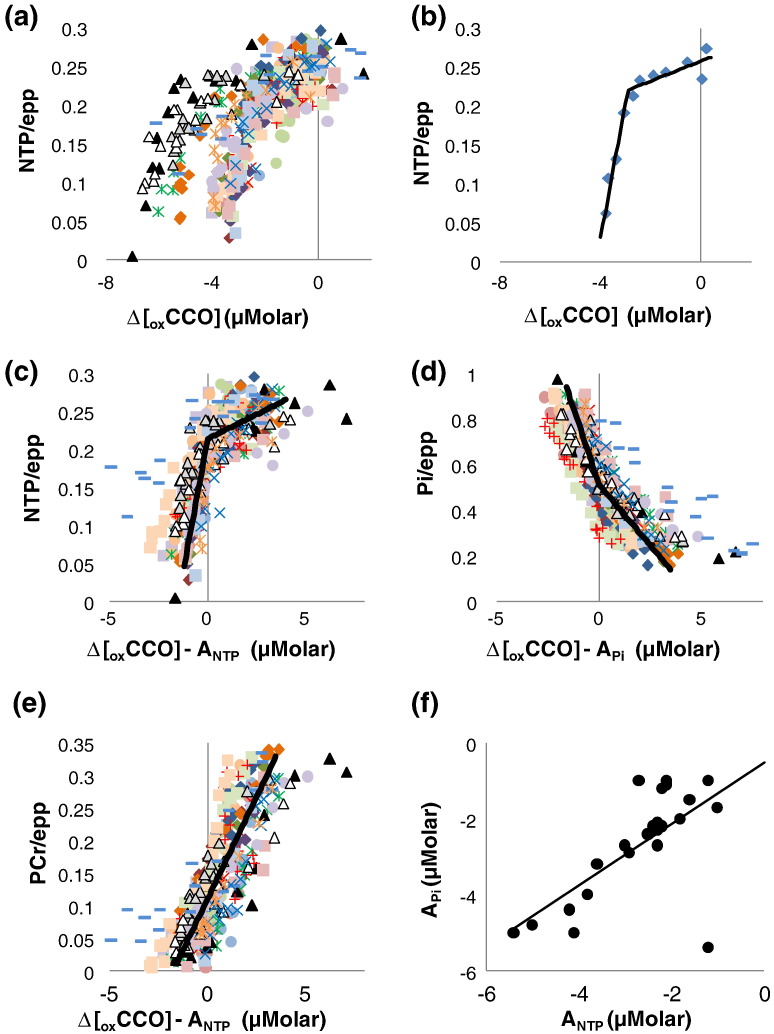
Correlations between ^31^P metabolite ratios and Δ[_ox_CCO]. (a) NTP/epp plotted against Δ[_ox_CCO] during HI (all data shown; different symbols for individual piglets). The relationship between NTP/epp and Δ[_ox_CCO] is similar for all piglets but for low NTP/epp the Δ[_ox_CCO] distribution clearly separates. (b) An example of a double-linear fit of an individual piglet. (c and d) NTP/epp and Pi/epp respectively plotted against Δ[_ox_CCO] − A (see Eq. (1)). Point A for each plot is now at 0 and all data are plotted on the same axes with group-mean data shown (thick black line). (e) PCr/epp vs Δ[_ox_CCO] − A_NTP_. (f) A_NTP_ plotted against A_Pi_ with the linear regression line shown. Independently fitted A_NTP_ is similar to A_Pi_.

Figs. 2a–b demonstrate the rationale for double-linear fitting NTP/epp and Δ[_ox_CCO]. Fig. 2a shows all data for each piglet demonstrating overall only a small NTP/epp decrease during initial Δ[_ox_CCO] decline up to a threshold after which NTP/epp decreases faster. This threshold point is defined by A in the double-linear model (see Eq. (1)). Fig. 2b shows the double-linear fit for a single piglet.

Each ^31^P ratio was plotted against Δ[_ox_CCO] for each subject and had both a linear and double-linear fit. The statistics from these fits are in Table 4. For presentation the Δ[_ox_CCO] axes in Figs. 2c–e were transposed such that Δ[_ox_CCO] = A was the origin. During early HI, Δ[_ox_CCO] declines but NTP/epp is effectively buffered until PCr/epp has fallen significantly (Figs. 2c and e); mean PCr/epp when Δ[_ox_CCO] = A_NTP_ (A for the NTP/epp fit) was 0.11 (0.04) (37% of baseline, p < 0.001 vs baseline, t-test). In Fig. 2c at Δ[_ox_CCO] = A_NTP_ NTP/epp had declined to only 0.21 (0.03) (86% of baseline; p < 0.001 vs baseline, t-test). The mean gradient of NTP/epp vs Δ[_ox_CCO] for Δ[_ox_CCO] > A_NTP_ was significantly lower than for Δ[_ox_CCO] < A_NTP_ (Table 4). As a percentage of the total fall in Δ[_ox_CCO] during HI (baseline Δ[_ox_CCO]–minimum Δ[_ox_CCO] during HI) Δ[_ox_CCO] had fallen by 71 (12) % when Δ[_ox_CCO] = A_NTP_.

**Table 4:** Regression analysis of Δ[_ox_CCO] plotted against each ^31^P MRS ratio during HI and Recovery. For data acquired during the HI period, summary data for both single linear and double-linear fits (see Eq. (1) in the section “Statistics”) are given in the table. Fitting with a double-linear model, slope a was significantly different to slope c for NTP/epp and Pi/epp. For data acquired during the Recovery period, summary data are given for single linear fits stratified according to outcome at 48 h. Fitted slopes were significantly lower in the Died group compared to Survivors.

^31^P MRS ratio	During HI						During Recovery		
	Single linear fit		Double linear fit				Single linear fit		
	Slope (μM^− 1^)	Mean R^2^	Slope (a) (μM^− 1^)	Slope (c) (μM^− 1^)	Δ[_ox_CCO] at point A (μM)	Mean R^2^	Outcome group	Slope (μM^− 1^)	Mean R^2^
NTP/epp	0.04 (0.01)	0.50 (0.18)	0.14 (0.09)	0.013 (0.017)^a^	− 2.7 (1.1)	0.79 (0.14)	Survivors	0.04 (0.02)	0.60 (0.27)
							Died	0.01 (0.01)^c^	0.23 (0.21)^b^
Pi/epp	− 0.14 (0.05)	0.79 (0.12)	− 0.27 (0.13)	− 0.11 (0.10)^a^	− 2.7 (1.3)	0.92 (0.07)	Survivors	− 0.14 (0.08)	0.71 (0.27)
							Died	− 0.03 (0.07)^c^	0.34 (0.25)^b^
PCr/epp	0.06 (0.02)	0.81 (0.17)	− 0.17 (1.3)	0.08 (0.06)	− 2.1 (1.1)	0.88 (0.13)	Survivors	0.07 (0.04)	0.68 (0.27)
							Died	0.02 (0.03)^c^	0.31 (0.24)^b^

T-test slope c vs slope a: ^a^p < 0.0001.

T-test died vs survivor group: ^b^p < 0.01, ^c^p < 0.001.

For Pi/epp, A_Pi_ (i.e. A for the Pi/epp fit) defines a threshold at which the mean rate of change of Pi/epp with Δ[_ox_CCO] increases towards the end of HI (Fig. 2d). At Δ[_ox_CCO] = A_Pi_, Pi/epp had doubled from baseline to 0.53 (0.10) (p < 0.001 vs baseline, t-test). The mean gradient of Pi/epp vs Δ[_ox_CCO] for Δ[_ox_CCO] < A_Pi_ was significantly higher than for Δ[_ox_CCO] > A_Pi_ (p < 0.0001, t-test).

The PCr/epp vs Δ[_ox_CCO] plot is shown in Fig. 2e with a single linear fit. There was no significant difference in the mean gradient of PCr/epp vs Δ[_ox_CCO] in the regions Δ[_ox_CCO] > A_PCr_ and Δ[_ox_CCO] < A_PCr_ (Fig. 2e). There were no significant differences between A_NTP_ and A_Pi_ for each subject (p > 0.9, paired t-test). Fig. 2f shows the A_NTP_ and A_Pi_ correlation. There were also no significant correlations between A_NTP_ and A_PCr_ or between A_Pi_ and A_PCr_.

### Relations between Δ[_ox_CCO] and MRS measures during Recovery

**Fig. 3:**
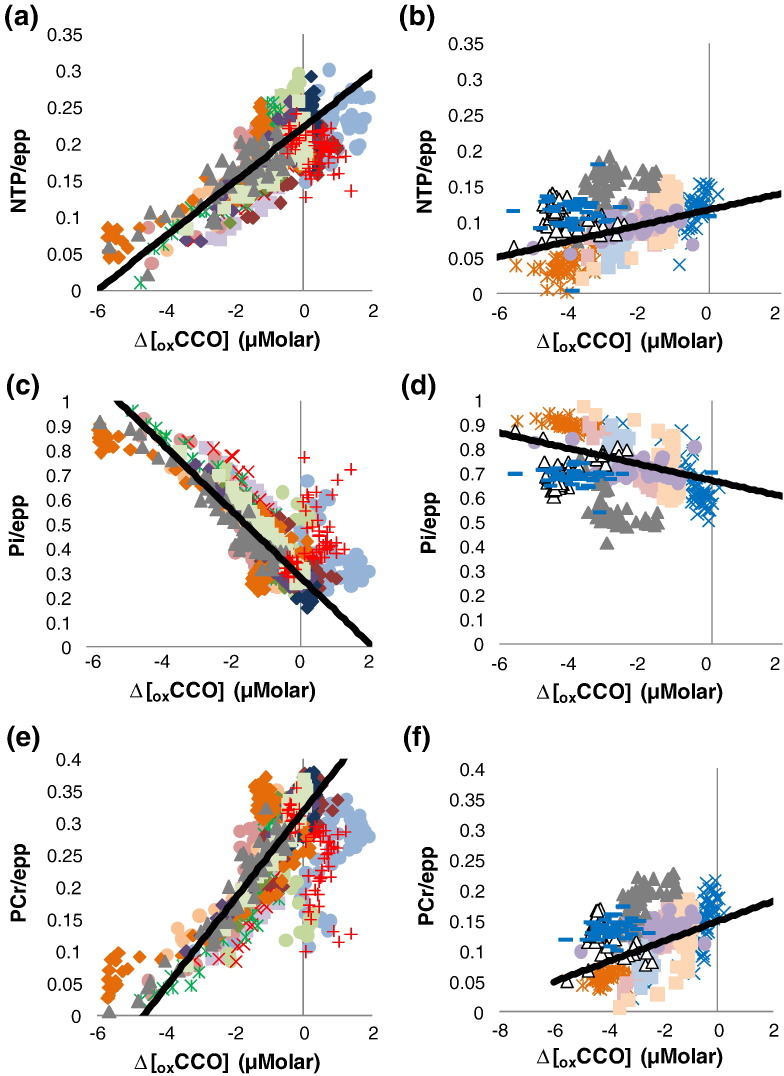
MRS ratios plotted against Δ[_ox_CCO] during Recovery. Data from all animals are shown. Data are stratified according outcome. Plots a (NTP/epp), c (Pi/epp) and e (PCr/epp)show data from those animals that survived to 48 h. Plots b (NTP/epp), d (Pi/epp) and f (PCr/epp) show data from animals that died before 48 h. Dark lines on each plot are overall linear regression lines calculated using data from all animals. Recovery slopes are significantly less steep for piglets dying before 48 h.

Fig. 3 shows MRS ratios plotted against Δ[_ox_CCO] during Recovery with died and survived groups plotted separately. Statistics from these fits are in Table 4. ^31^P ratio and Δ[_ox_CCO] recovery were significantly lessened for piglets dying before 48 h (Table 4). Overall ^31^P ratio vs Δ[_ox_CCO] linear-fit gradients were significantly larger in the survivor group. ^31^P ratio vs Δ[_ox_CCO] correlations were also weaker (smaller mean R^2^) for piglets dying before 48 h (Table 4; p < 0.01 for all comparisons; t-test).

## Discussion

We used ^31^P MRS to study brain high-energy phosphates and NIRS to study the oxidation status of cytochrome oxidase in the brain with the aim of elucidating the relationships between these measurements during and after HI. We demonstrated that the extent of recovery of Δ[_ox_CCO] and ^31^P MRS metabolite ratios were associated during the early period of recovery post HI. During HI, PCr/epp appears to decline linearly with Δ[_ox_CCO]. The majority of the decline in NTP/epp occurred after PCr/epp has decreased to approximately 1/3rd of its baseline value. Thus there was a clear threshold, A_NTP_, in the NTP/epp vs Δ[_ox_CCO] plots where the gradient of the regression line changed significantly. Similarly, there was a threshold point, A_Pi_, where the gradient of the Pi/epp vs Δ[_ox_CCO] curve increased significantly; A_NTP_ and A_Pi_ correlated significantly. During recovery, animals that survived to 48 h demonstrated a more complete recovery of ^31^P MRS ratios and Δ[_ox_CCO]; animals that died or were terminated prior to 48 h had weaker recovery of both ^31^P MRS ratios and Δ[_ox_CCO].

### The model

The piglet asphyxia model used in the current study has strong pre-clinical value; it allows intensive care to be applied as it would be in an intensive care setting in the clinic, thus maintaining metabolic and physiological homeostasis. The model was developed to replicate the delayed appearance of abnormalities observed on ^31^P MRS spectra in human neonates acquired in the early period after birth (Hope et al., 1984). The data acquired from the model has relevance to the human newborn with hypoxic–ischaemic encephalopathy. MRS biomarkers are known to change during and after HI (Lorek et al., 1994), serve as linking biomarkers in infants with HIE (Cheong et al., 2006) and are used as surrogate outcome measures in neuro-protection trials. Furthermore, the timing and evolution of cerebral ^31^P and ^1^H MRS biomarkers are similar in the piglet following HI and the regional pattern of injury is similar to that observed in humans and in primate models (Myers, 1975). Importantly, several neuro-protection studies performed in the piglet model have provided proof of concept data for subsequent translational studies in newborn babies in the clinic, for example therapeutic hypothermia (now standard therapy for HIE) (Tagin et al., 2012), xenon augmented hypothermia (Faulkner et al., 2011), melatonin (Robertson et al., 2012a) and amiloride (Robertson et al., 2012b). This study however furthers our understanding in that we define particular thresholds for NTP below which the correlation between NTP and CCO changes dramatically.

### The double-linear model

Visual inspection of the data suggested that the relationship between NTP/epp and Δ[_ox_CCO] during HI was similar in each subject in terms of the pattern of change but heterogeneous in terms of the absolute values of Δ[_ox_CCO]. The double-linear model was used to extract the general features of the relationship between NTP/epp and Δ[_ox_CCO] rather than to directly model any specific features of the underlying bio-chemistry. Nevertheless the parameter A in the model does define a specific reference position on the Δ[_ox_CCO] axis. The raw data in Fig. 2a demonstrate well the heterogeneity of the Δ[_ox_CCO] signal across the subject group. By transposing each dataset such that A = 0, as illustrated in Fig. 2c, the general pattern of the relationship between NTP/epp and Δ[_ox_CCO] can be more easily seen. This model was extended to investigate the relations between Pi/epp and Δ[_ox_CCO] and between PCr/epp and Δ[_ox_CCO].

Additionally, each dataset was fitted with a linear function. In general, care must be taken to avoid over-specifying the model function; usually, when fitting two functions to the same data, it is feasible to perform a statistical comparison to determine whether the more complex function yields a better fit to the data. However, in this case, the linear regression line is simply equivalent to a special-case solution to the double linear model where the gradient is identical either side of point A. Thus the R^2^ value for the double-linear model fit will always be ≥ R^2^ from a simple linear regression. A statistical comparison of the model function fits (for example by F-test of the residuals to the regression lines) is therefore likely to be meaningless. For the NTP/epp vs Δ[_ox_CCO] and the Pi/epp vs Δ[_ox_CCO] datasets there was a significant change in the gradient of the regression line at point A. Furthermore, there was a significant correlation between A_NTP_ and A_Pi_. This is taken as evidence that A_NTP_ and A_Pi_ have some physical significance in these data and is our justification for considering the fits to the double-linear model when discussing these data in further detail below. In contrast, for the PCr/epp vs Δ[_ox_CCO] datasets there was no significant change in gradient at A_PCr_ and no correlation between A_PCr_ and either A_NTP_ or A_Pi_. Thus the results of using a simple linear regression to these data are considered in the discussion below. For completeness the results of both the double-linear model and simple linear model fits are included in Table 4.

### Response of the brain during HI

For the analysis of the data acquired during HI, all subjects were analysed as a single group. There was no difference between the subjects at baseline when stratified according to outcome group (see Table 2) and no justification for stratifying subjects at this stage.

A model of the biochemistry of the piglet brain during anoxia has been previously developed to simulate NIRS and MRS data (Moroz et al., 2012) and this informs much of the following discussion of the experimental changes seen in this work. Δ[_ox_CCO] is closely linked to the production of ATP and, by extension, the ^31^P MRS measurement of NTP/epp. We interpret a decreased Δ[_ox_CCO] as impairment of the electron transport chain (ETC) which in turn results in failure of ATP production through ATP synthase. The double-linear model defines the point A_NTP_ at which there is a threshold change in the relationship between NTP/epp and Δ[_ox_CCO]. During the initial period of HI, Δ[_ox_CCO] declines and so does ATP synthase. During this period, ATP levels are initially buffered through the creatine kinase reaction leading to a decline in PCr (Lawson and Veech, 1979). At the A_NTP_ point, NTP/epp has reduced only slightly whereas PCr/epp is severely depleted at approximately 1/3rd of its baseline value. Eventually, after the A_NTP_ point, the depletion of PCr causes ATP to decline and therefore NTP/epp declines faster with Δ[_ox_CCO]. The model of Moroz, along with experimental data (Korzeniewski and Zoladz, 2001), suggests that the reaction rate for ATP hydrolysis remains high until ATP concentrations are severely depleted. Thus Pi is produced as a by-product and, because ATP synthase is failing, Pi/epp increases. At the A_Pi_ point, not significantly different from A_NTP_, the slope of the Pi/epp vs Δ[_ox_CCO] plot increases. Presumably, as NTP/epp starts to fall quickly at this point, there is now little production of ATP via ATP synthase. The increase in the slope of Pi/epp vs Δ[_ox_CCO] may therefore reflect the continuing demand for ATP (ATP is not yet severely depleted at the A_NTP_ point) along with now much reduced re-combination of Pi into ATP via ATP synthase. We speculate that the A_NTP_ and A_Pi_ points represent the point at which ATP manufacture via all pathways is almost completely suppressed. Furthermore, at this point over 2/3rds of the eventual reduction of Δ[_ox_CCO] has occurred suggesting that the majority of the reduction in Δ[_ox_CCO] occurs during the period where ATP manufacture via ATP synthase declines. The change in the slopes of the NTP/epp vs Δ[_ox_CCO] and the Pi/epp vs Δ[_ox_CCO] regression lines and the strong correlation between A_NTP_ and A_Pi_ are further evidence that the decline in Δ[_ox_CCO] represents the decline in ETC activity in this model.

### Response of the brain during recovery

During recovery from HI, the electron transport chain activity recovers and Δ[_ox_CCO] starts to recover as oxidative phosphorylation resumes. This leads to Pi reduction as it combines with ADP to produce ATP. The production of ATP causes PCr reserves to be restored via creatine kinase and recovery of both NTP/epp and PCr/epp is observed on ^31^P MRS.

Nine piglets showed poor recovery during the first hour post HI and had to be terminated prior to 48 h post HI because of the extent of their injury. The HI in this experiment is severe and is designed to induce brain injury. The parameters of the HI were carefully controlled and reproduced in each experiment. The level of NTP was monitored in real time during HI and the FiO_2_ adjusted during Titration to ensure that β-NTP peak height was held between 30% and 40% of its baseline value. Despite a similar level of hypoxia–ischaemia in our model, there is a heterogeneous response to this insult. Many factors including aetiology, extent of hypoxia and ischaemia, maturational stage of the brain, regional cerebral blood flow and general health of the subject all influence the response to injury. This is similar to the heterogeneous response to injury seen in human fetuses and babies. After HI, Δ[_ox_CCO], PCr/epp and NTP/epp remained lower and Pi/epp remained higher in these animals than in survivors. ATP levels in early recovery are dependent on the severity of HI with lower ATP levels correlating with longer periods of ischemia (Nishijima et al., 1989, Phillis et al., 1996). Indeed, the response of NTP/epp during and for 1 h after HI has previously been used as a measure of insult severity (O'Brien et al., 2006). Furthermore, ^31^P metabolite ratios 2 h post HI can be used as early markers of the likely extent of injury with increased Pi/epp and decreased PCr/epp and NTP/epp correlating with poor outcome in the piglet at 48 h (Cady et al., 2008). The MRS ratios measured during the early recovery period are consistent with early markers of adverse outcome.

### Factors affecting the measurements of ^31^P MRS and NIRS biomarkers

MRS was performed using a simple pulse-acquire sequence, and the signal was localised to the brain by using a surface coil in transmit and receive mode. The dimensions of the coil limit the volume over which MRS signals are acquired and so effectively localise the signal acquisition to the brain. NIRS data were acquired in transmission mode with optodes positioned on the right and left hand sides of the head at approximately the middle part of the brain. The use of transmission mode for NIRS is a significant development compared to previous studies (Cooper and Springett, 1997, Springett et al., 2000a). The surface coil used for MRS was sufficiently large to acquire signals from the deep grey matter nuclei as well as the superficial structures of the brain. Computational simulations suggest that transmission mode NIRS affords greater sensitivity to deeper structures (Gunadi and Leung, 2011) than does reflection mode. The data from both modalities represent an average over both superficial and deep brain structures. Although the positioning of the coil and optodes was arranged such that data were acquired from similar volumes of tissue, the weights of contributions from different brain structures will be different—^31^P data are more weighted to superficial structures given the positioning of coil on the top of the head.

^31^P MRS data are given as metabolite peak-area ratios. Referencing to epp allows separate interpretation of PCr, Pi and NTP changes and there is evidence (Cady et al., 2008) that these ratios are more sensitive to abnormalities than the PCr/Pi ratio used in many previous studies. However, the ^31^P biomarkers are not direct concentration measurements. Interpreting changes in metabolite/epp ratios as equivalent changes in the concentration of the metabolite relies on an additional assumption that the net contributions of PCr, Pi and NTP to the total visible phosphorus pool is unchanged through the course of the experiment.

The NIRS measurements in this work are of relative changes therefore we cannot directly estimate tissue oxygen saturation. Previously Springett et al. (2000b) estimated tissue saturation after calibrating the system to complete anoxia. However, this was not within the scope of this work. Given that Δ[_ox_CCO] is a measurement only of the change in oxidation state of CCO, there is no information about the absolute value of [_ox_CCO] at baseline and no *a priori* knowledge as to the concentration of mitochondria in the brain. The optical set-up, optical algorithm and experimental methodology did not allow us to measure the absolute baseline oxidation state of the CCO. Previously a methodology has been described to quantify absolute deoxyhaemoglobin (Matcher and Cooper 1994). Recently the absolute oxidation of CCO has been using light in the visible spectrum (Sakata et al., 2012). We are actively pursuing the development of new instrumentation to quantify independently the absorption and scattering and hence, using the independent spectra of the oxidation and the reduced CCO, to quantify its absolute redox state (Tachtsidis et al., 2010).

### Factors affecting Δ[_ox_CCO]

Mathematical modelling suggests that an increased substrate supply of electrons reduces [_ox_CCO] and increased oxygen supply oxidises [_ox_CCO] (Banaji, 2006). Experimentally, other factors such as pH and mitochondrial inhibitors may explain other small changes in Δ[_ox_CCO] (Cooper and Springett, 1997). During HI, decreased substrate supply and reduced oxygenation would have an opposite effect on the oxidation states of electron transport chain intermediates and, consequently, on Δ[_ox_CCO]. In the fetal sheep, Δ[_ox_CCO] has been observed to increase during 25 min of complete occlusion of the umbilical cord (Bennet et al., 2007) and during occlusion of maternal common internal iliac artery for 1 h (Newman et al., 2000). Bennet et al. speculate that increased Δ[_ox_CCO] during occlusion is a result of reduced substrate supply of electrons during occlusion suggesting a rapid decrease in metabolic activity. In the naïve healthy piglet brain, small changes in oxygen delivery do not induce concurrent changes in Δ[_ox_CCO] (Quaresima et al., 1998); reductions in Δ[_ox_CCO] typically reflect larger scale reductions in oxygen supply. Furthermore, Tsuji et al. (1995) have shown in the piglet that hypoxia with 12% and 8% FiO_2_ produced slight increases in Δ[_ox_CCO] whereas only for more severe hypoxias did Δ[_ox_CCO] reduce. Our observation of a profound reduction in Δ[_ox_CCO] during HI is consistent with previous observations in the piglet (Springett et al., 2000a). The reasons for the differences in the data from pigs and sheep are not clear but data from individual models appear consistent.

### Interpretation of results

The NIRS and ^31^P MRS data acquired in this study are averaged measurements on the whole brain. The evolution of the ^31^P metabolite ratios during HI has been described in the section “Response of the Brain during HI” in terms of the biochemistry that is happening at a cellular level despite that fact that the data originate from a heterogeneous population of cells. We speculate that this description is possible because during acute HI the cells are responding similarly and approximately simultaneously to a global stimulus such that the average signals over the bulk tissue represent a good approximation to the changes occurring at a single cellular level. The relationships between ^31^P metabolite ratios and Δ[_ox_CCO] therefore provide some insight into the significance of Δ[_ox_CCO] decline during HI, in particular that this likely represents the decline in ETC activity. In contrast, recovery from HI is likely to be heterogeneous (Vannucci et al., 2004). It is likely that there will be different populations of cells that differ in both the extent and rate of their recovery. Thus, the observed pattern of recovery of ^31^P metabolite ratios is likely to be a poor representation of the pattern of change at a single cellular level. Similarly, the observed relationships between ^31^P metabolite ratios and Δ[_ox_CCO] are less likely to accurately describe the true relationships at a single cellular level. Nevertheless, these data may provide an index of the averaged extent of recovery in the brain. Persisting reduced NTP/epp in the Recovery period after restoration of oxygen supply suggests synthesis of ATP is either impaired or absent. Thus this implies either impaired mitochondrial function or cell death. In the poor outcome group there was poor recovery of both Δ[_ox_CCO] and NTP/epp. This suggests that mitochondrial metabolism is not now generating ATP and that, in this model, continued mitochondrial impairment after HI may be the cause of continued suppression of Δ[_ox_CCO].

### Translation to the clinic

The NIRS system used in this study was in-house developed and adapted to monitor human neonates. It is based on using multiple wavelengths (not just two or three discreet wavelengths) to improve the precision of the measurement (Kolyva et al., 2012, Tisdall et al., 2008b). The instrumentation operation and applicability is similar to other commercial NIRS systems. Movement artefacts can affect the signals therefore careful consideration is needed regarding the application of the optode holder to the head. Measurement of the oxidation state of CCO has the potential to provide information about the severity of HI and whether the brain is recovering in the early period after birth. However, it is also true that the oxidation state of naïve brain before the insult is not known and hence the comparison with the injured oxidation state cannot be made. The development of absolute measurement of _ox_CCO will simplify the clinical interpretation. Nevertheless, in this study we demonstrated that _ox_CCO measurement is a potential marker of metabolism in the brain independent from the haemoglobin/oxygenation measurement. Measurement of the trend of Δ[_ox_CCO] over time after the injury is likely to indicate the degree of development of SEF and hence will be useful to collect to determine the extent of recovery of the brain.

## Conclusions

We have demonstrated the monitoring *in*-*vivo* of the metabolic events during and following HI with high temporal-resolution. During HI there are significant correlations between ^31^P MRS ratios and Δ[_ox_CCO]. Both NTP/epp and Pi/epp exhibit a significant change in their relations with Δ[_ox_CCO] at the point when PCr/epp becomes severely depleted. During recovery those animals that survived to 48 h demonstrated more complete recovery of ^31^P MRS ratios and Δ[_ox_CCO] than those who subsequently develop secondary energy failure.
